# *Lavandula angustifolia* Extract Improves the Result of Human Umbilical Mesenchymal Wharton's Jelly Stem Cell Transplantation after Contusive Spinal Cord Injury in Wistar Rats

**DOI:** 10.1155/2016/5328689

**Published:** 2016-02-14

**Authors:** Kayvan Yaghoobi, Gholamreza Kaka, Korosh Mansouri, Shaghayegh Davoodi, Seyed Homayoon Sadraie, Seyed Ruhollah Hosseini

**Affiliations:** ^1^Neuroscience Research Center, Baqiyatallah University of Medical Sciences, Tehran 19568-37173, Iran; ^2^Department of Physical Medicine and Rehabilitation, Iran University of Medical Sciences, Tehran, Iran; ^3^Department of Anatomy, School of Medicine, Baqiyatallah University of Medical Sciences, Tehran, Iran

## Abstract

*Introduction.* The primary trauma of spinal cord injury (SCI) results in severe damage to nervous functions. At the cellular level, SCI causes astrogliosis. Human umbilical mesenchymal stem cells (HUMSCs), isolated from Wharton's jelly of the umbilical cord, can be easily obtained. Previously, we showed that the neuroprotective effects of* Lavandula angustifolia* can lead to improvement in a contusive SCI model in rats.* Objective.* The aim of this study was to investigate the effect of* L. angustifolia* (Lav) on HUMSC transplantation after acute SCI.* Materials and Methods.* Sixty adult female rats were randomly divided into eight groups. Every week after SCI onset, all animals were evaluated for behavior outcomes. H&E staining was performed to examine the lesions after injury. GFAP expression was assessed for astrogliosis. Somatosensory evoked potential (SEP) testing was performed to detect the recovery of neural conduction.* Results.* Behavioral tests showed that the HUMSC group improved in comparison with the SCI group, but HUMSC + Lav 400 was very effective, resulting in a significant increase in locomotion activity. Sensory tests and histomorphological and immunohistochemistry analyses verified the potentiation effects of Lav extract on HUMSC treatment.* Conclusion.* Transplantation of HUMSCs is beneficial for SCI in rats, and Lav extract can potentiate the functional and cellular recovery with HUMSC treatment in rats after SCI.

## 1. Introduction

There are approximately 200,000 spinal cord injuries (SCIs) annually in the United States, the vast majority of which are caused by motor vehicle accidents [1]. SCI can result in severe damage to the motor, sensory, and autonomic nervous systems and their function and may lead to paraplegia and severe disabilities [2].

The pathogenesis of SCI after the primary trauma plays an important role in initial tissue disruption, and the subsequent series of secondary cellular processes can lead to long-term spinal deficits [3, 4]. Increased oxidative stress [5] and activation of redox transcription factors, as well as elevated expression of inflammatory mediators, may play some of the most important roles [6] in promoting secondary injuries after SCI. SCI may be followed by the degeneration of axons, the loss of neurons as well as glia, and demyelination around the lesion site. Axonal regeneration in the central nervous system (CNS) is impeded partly by myelin-associated inhibitors [7, 8] and the formation of a postlesion scar barrier [9]. The extent of intrinsic cell renewal alone [10], even after the application of mitogenic agents, such as epidermal growth factor and fibroblast growth factor-2 [11, 12], is not sufficient to allow substantial recovery following SCI [13]. Therefore, therapeutic strategies such as exogenous cell replacement should be considered. Human umbilical mesenchymal cells (HUMSCs) from Wharton's jelly possess stem cell properties [14] and express type I MHC molecules, MSC markers (SH2 and SH3), and adhesion molecules (CD44 and CD105), but not type II MHC and hematopoietic markers (CD34 and CD45) [15]. HUMSCs from Wharton's jelly are primitive, uncontaminated, and immunotolerable [15] and are a low-cost source of stem cells that can be easily obtained and propagated in culture without invasive medical procedures or ethical issues [16]. These cells can be induced to form other cell lineages, such as neurons and glial cells [17, 18].

HUMSCs are also capable of differentiating into osteogenic, chondrogenic, adipogenic, and myogenic cells* in vitro* [19]. HUMSCs might be a good stem cell source for transplantation [20]. There is ample evidence that stem cell therapy could be effective in SCI [21], but we need a strategy to potentiate these stem cell transplantation results. As there has been some interest in finding natural agents that may help to prevent the inflammation and degeneration of neural cells in SCI, one of the well-known herbal drugs that has demonstrated antioxidant effects is Lavender. Lavender, or* Lavandula angustifolia* Mill. (Lamiaceae), commonly known in Iran as “Ostokhoddous,” is a widely distributed aromatic herb [22]. It has been used widely for nervous system problems in Iranian traditional medicine [23], and it has recently been demonstrated to have important effects on the central and peripheral nervous systems, including anti-inflammatory, antiapoptosis, antioxidant, antimutant, and neuroprotective effects [24]. Gas chromatography-mass spectrometry analysis extraction of* L. officinalis* L. from Urmia, Iran, showed totals of 60 and 100 compounds, respectively, in 96% and 70% ethanol solvent extractions [25]. The most abundant constituents observed in ethanol 96% extraction included ethane (29.80%), methanecarboxylic acid (9.01%), p-vinylguaiacol (4.45%), pentadecanoic acid (3.67%), and dimethylamine, N,N-dimethyl methanesulfonamide (2.06%) [25]. Yuanyuan et al. identified 17 compounds in lavender from Xinjiang, China, with linalool (44.54%), geraniol (11.02%), lavandulyl acetate (10.78%), 3,7-dimethyl-2,6-octadien-1-ol (10.35%), and isoterpineol (6.75%) as the main components [26]. It is known that linalool is responsible for important therapeutic effects [27, 28]. Each of these constituents can vary significantly in oils derived from different cultivars, and variations can affect the medical properties; therefore, this study aimed to assess the effect of* L. angustifolia* extract from Iran on SCI treated with HUMSCs. Previously, we showed that the effective dose of* L. angustifolia* was 400 mg/kg in an SCI contusive model, and this* Lavandula* extract was effective at improving behavioral, sensory, and cellular function after SCI. We hypothesized that* L. angustifolia* may play a role in preventing the harmful effects and neural damage triggered by SCI, promoting axonal regeneration, and potentiating stem cell transplantation effects on behavioral, sensory, and cellular function after SCI. The aim of this study was to assess the effect of* L. angustifolia* extract on the outcome of transplantation of HUMSCs from Wharton's jelly after contusive SCI in Wistar rats.

## 2. Materials and Methods

### 2.1. Drug Treatments and Experimental Outline

Sixty rats were divided into eight groups as follows: group I: intact (*n* = 6); group II: sham-operated/saline (*n* = 6); group III: control 1, subjected to SCI (*n* = 7); group IV: Lav 400 mg/kg (*n* = 8); group V: control 2, subjected to HUMSC (*n* = 7); group VI: SCI treated with HUMSC + Lav 100 mg/kg (*n* = 8); group VII: SCI treated with HUMSC + Lav 200 mg/kg (*n* = 10); and group VIII: SCI treated with HUMSC + Lav 400 mg/kg (*n* = 8). Lav and saline, respectively, were injected intraperitoneally in the Lav and sham groups starting one day after injury and then daily for 14 days. We used the SCI group as control 1 for comparison with the HUMSC group, and we used the HUMSC group as control 2 for comparison with the HUMSC + Lav-treated groups. As we determined the effective dose (400 mg/kg) of* L. angustifolia* in SCI in our previous study, we used that dose for this study.

### 2.2. Intraspinal Cord HUMSC Transplantation: Preparation of HUMSCs

After considering all ethical aspects and receiving permission from the parents, fresh umbilical cords were aseptically collected from full-term infants after cesarean section. The study adhered to the policies of Baqiyatallah Hospital and received approval from the Ethical Committee of Baqiyatallah University of Medical Sciences, Tehran, Iran. The experiment followed the Iranian Ministry of Health and Medical Education's guidelines for laboratory animals. The fresh human umbilical cords were collected in Hanks' Balanced Salt Solution (HBSS) (Gibco, USA) at 4°C. Following disinfection in 75% ethanol for 30 sec, the umbilical cord vessels were cleaned off while still in HBSS. The mesenchymal tissue in Wharton's jelly was then diced into cubes of about 0.5 cm each and centrifuged at 250 g for 5 min. Following removal of the supernatant fraction, the precipitate (mesenchymal tissue) was washed with serum-free DMEM (Gibco) and centrifuged at 250 g for 5 min. Following aspiration of the supernatant fraction, the precipitate (mesenchymal tissue) was treated with collagenase at 37°C for 18 hours, washed, and further digested with 2.5% trypsin (Gibco) at 37°C for 30 min. Fetal bovine serum (FBS; Hyclone, USA) was then added to the mesenchymal tissue to stop trypsinization. The cells were incubated for 2-3 days until the cells reached confluency, and the culture was repeated for four passages (P4), with one week for each passage. At P4, the cells were checked for the properties of bone marrow stem cells (BMSCs) using fibronectin (+), CD44 (+), CD90 (+), and CD45 (−) immunostaining. The dissociated mesenchymal cells were further dispersed in 10% FBS-DMEM and counted under a microscope with the aid of a hemocytometer (Figure 5). The mesenchymal cells were then used directly for cultures or stored in liquid nitrogen for later use. These cells were then separately transplanted into three positions on the injured rat spines 24 hours after injury.

### 2.3. Intraspinal Transplantation of HUMSCs

The animals were reanesthetized as described before, and the laminectomy site was reexposed. The sham group animals were injected 24 h after laminectomy with 9 *μ*L of normal saline using a 10 *μ*L Hamilton syringe. The HUMSC-treated group was injected 24 h after injury. The marked HUMSCs (3 × 10^5^ cells/*μ*L) with 5-bromo-2′-deoxyuridine (BrdU) in 9 *μ*L of normal saline were sucked into a Hamilton syringe and then injected slowly at a rate of 0.25 *μ*L/min with a microinjector, into three separate locations of the lesion area (epicenter, distal, and proximal) at a depth of 1.2 mm.

The HUMSCs were previously labeled with BrdU in order to facilitate identification of the cells within the subsequent histological specimens. After a 5 min delay, the fascia were sutured. The animals were kept on warming pads (37°C) until recovery, and after recovery they were returned to their previous cages. All procedures were done under aseptic conditions with complete anesthesia.

## 3. Results

### 3.1. Effects of* L. angustifolia* Extract on Locomotor Recovery after SCI Treatment with HUMSCs

While the SCI resulted in immediate paraplegia (loss of hindlimb movement), there were no significant differences in locomotion scores (BBB) of the sham-operated rats in comparison with the intact animals. HUMSC treatment significantly improved locomotor function compared to the control group. However, when we added intraperitoneal Lav treatment (100, 200, or 400 mg/kg) one day after injury, locomotor function was significantly improved compared to the control, HUMSC, and Lav groups. Application of two-way ANOVA showed significant interaction between variables, such as HUMSC therapy, Lav treatment (100, 200, and 400 mg/kg), and time (*F*(81,585) = 57.27, *P* < 0.0001).

**Figure 1:**
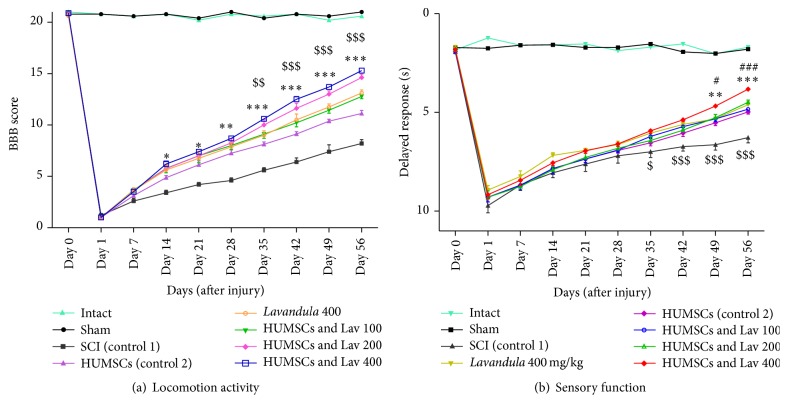
Administration of* L. angustifolia* extract improves motor and sensory function impairment in the rat spinal cord contusion model treated with HUMSCs. Administration of* L. angustifolia* extract (i.p.) daily for 14 consecutive days after injury significantly improved BBB scores (a) and sensory function (with decreased delayed response in the hot-water test) in HUMSCs treated animals (b). Data are represented as mean ± SEM. (a) ^*∗*^, ^*∗∗*^,  and ^*∗∗∗*^ show significant differences of BBB scores between HUMSCs, HUMSCs + Lav 100, 200, and 400, and SCI group (control 1) (*P* < 0.05, *P* < 0.001, and *P* < 0.0001, resp.). ^$^, ^$$^, and ^$$$^ show significant differences of BBB scores between HUMSCs + Lav 100, 200, and 400, and HUMSCs treated group (control 2) (*P* < 0.05, *P* < 0.001, and *P* < 0.0001, resp.). (b) ^*∗∗*^ and ^*∗∗∗*^ show significant differences of sensory function between HUMSCs and SCI group (control 1) (*P* < 0.001 and *P* < 0.0001, resp.). ^#^ and ^###^ show significant differences of sensory function between HUMSCs + Lav 400 and HUMSCs treated group (control 2) (*P* < 0.05 and *P* < 0.0001, resp.). ^$^, ^$$^, and ^$$$^ show significant differences of sensory function between HUMSCs + Lav 400 and SCI group (control 1) (*P* < 0.05, *P* < 0.001, and *P* < 0.0001, resp.).

Application of Bonferroni's post hoc multiple-comparison test revealed significant improvement in motor function following HUMSC therapy from day 14 after injury (*P* < 0.01) until day 56 (*P* < 0.001). Adding Lav extract improved the BBB scores in comparison with the SCI group from days 14 through 56 after injury (*P* < 0.001) and also improved BBB scores in comparison with the HUMSC group on day 56 after injury (*P* < 0.01) at the dose of 100 mg/kg. Lav extract at doses of 200 and 400 mg/kg were more effective and resulted in more improved BBB scores in comparison with the HUMSC group after day 35 at both doses (*P* < 0.001). However, the Lav dose of 400 mg/kg with HUMSCs also resulted in better BBB scores on days 14 (*P* < 0.05), 21 (*P* < 0.05), and 28 (*P* < 0.01) after injury. HUMSCs with Lav extract potentiated the BBB scores in comparison with the SCI, Lav, and HUMSC groups (Figure 1(a)).

### 3.2. Effects of* L. angustifolia* Extract on Sensory Recovery after SCI Treated with HUMSCs from Wharton's Jelly

Statistical evaluations revealed that the mean latency time of response to painful stimuli was significantly decreased in the HUMSC group versus the control group. However, when we added intraperitoneal Lav treatment (100, 200, and 400 mg/kg) one day after injury, sensory recovery was significantly improved compared to the control, HUMSC, and Lav groups. Two-way ANOVA showed significant interactions between variables, including SC, Lav dose (100, 200, or 400 mg/kg), and time (*F*(81,621) = 20.22, *P* < 0.0001).

Application of Bonferroni's post hoc multiple-comparisons test revealed significant improvement in sensory function following HUMSC therapy in comparison with the SCI group on days 49 (*P* < 0.01) and 56 (*P* < 0.001) after injury. However, when we added intraperitoneal Lav treatment (100, 200, or 400 mg/kg) one day after injury, sensory function was significantly improved compared to the control, HUMSC, and Lav groups, separately.

There were no significant differences between the HUMSC group and the HUMSC + Lav 100 and HUMSC + Lav 200 groups, but there was significant improvement in sensory function in the SC + Lav 400 group on days 49 (*P* < 0.01) and 56 (*P* < 0.001) after injury (Figure 1(b)).

### 3.3. Effects of* L. angustifolia* Extract on Electrophysiological Recovery after SCI Treatment with HUMSCs from Wharton's Jelly

Statistical analysis showed that the mean recruitment indexes were increased significantly for the left (*F*(9,64) = 13.63, *P* < 0.0001) and right (*F*(9,64) = 20.44, *P* < 0.0001) hindlimbs in the Lav + HUMSC groups versus the control HUMSC and SCI groups.

**Figure 2:**
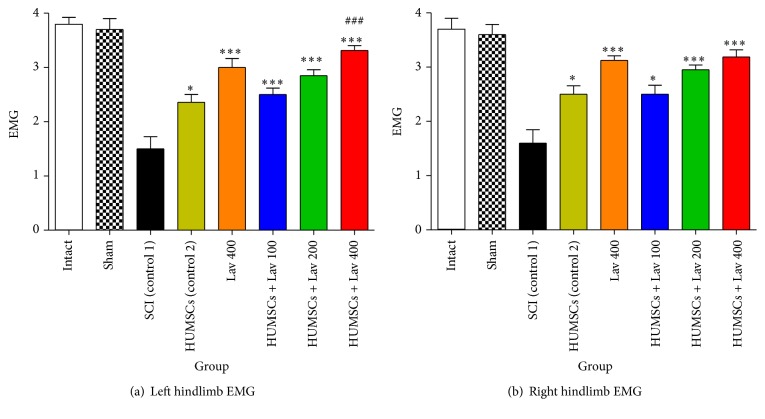
Stem cell therapy and administration of* L. angustifolia* improved locomotor and EMG impairment in the rat spinal cord contusion model. Intraperitoneal administration of* L. angustifolia* extract daily for 14 consecutive days after injury significantly improved the EMG results in left (a) and right hindlimbs (b) in HUMSCs treated groups. Data are represented as mean ± SEM. (a) ^*∗*^ and ^*∗∗∗*^ show significant differences between HUMSCs + Lav 100, 200, and 400 compared to the SCI group (control 1) (*P* < 0.05 and *P* < 0.0001, resp.). ^###^ shows significant differences between HUMSCs + Lav 400 (control 2) and HUMSCs (*P* < 0.0001). (b) ^*∗*^ and ^*∗∗∗*^ show significant differences between HUMSCs + Lav 100, 200, and 400 compared to the SCI group (control 1) (*P* < 0.01 and *P* < 0.0001, resp.).

Application of Bonferroni's post hoc multiple-comparisons test, as well as Bartlett's test for equal variances, revealed significant improvement in electrophysiological activity of the right hindlimb following stem cell therapy and HUMSC + Lav 100, HUMSC + Lav 200, and HUMSC + Lav 400 (*P* < 0.0001) and the left hindlimb following stem cell therapy in the HUMSC + Lav 100, HUMSC + Lav 200, and HUMSC + Lav 400 (*P* < 0.0001) treatment groups compared with the SCI group (Figure 2). Although there were some improvements in the HUMSC + Lav treatment groups in comparison with the control HUMSC group, these improvements were not significant in the right hindlimb but were significant in the HUMSC + Lav 400 group left hindlimbs in comparison with HUMSC therapy alone (*P* < 0.0001). The most-recovered hindlimb results were in the HUMSC + Lav 400 group, as there were no significant differences between the sham-operated left and right hindlimb EMG results versus the HUMSC + Lav 400 group's results.

### 3.4. Effects of* L. angustifolia* Extract on Histomorphological Findings after SCI Treatment with HUMSCs from Wharton's Jelly

**Figure 3:**
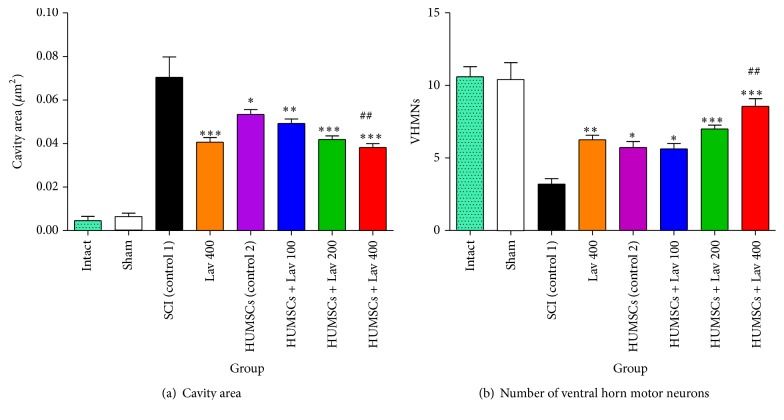
HUMSCs transplantation and administration of* L. angustifolia* improved histomorphological results, decreased cavity area (a), and increased the number of VHMNs (b) in the rat spinal cord contusion model. Intraperitoneal administration of* L. angustifolia* extract daily for 14 consecutive days after injury significantly improved histomorphological results including cavity area and VHMNs results. Data are represented as the mean ± S.E.M. ^*∗*^, ^*∗∗*^, and  ^*∗∗∗*^ show significant differences of HUMSCs, HUMSCs + Lav 100, 200, and 400 compared to SCI (control 1) group (*P* < 0.05, *P* < 0.0001, and *P* < 0.0001, resp.). ^##^ shows significant differences between HUMSCs + Lav 400 and HUMSCs (control 2) (*P* < 0.001). Cavity area significantly decreased in HUMSCs and HUMSCs + Lav-treated groups (a) (Per 35625 *μ*m^2^). Number of ventral horn motor neurons significantly increased in HUMSCs and HUMSCs + Lav-treated groups (b) (Per 5700 *μ*m^2^).

Statistical evaluations revealed that the mean cavity size was significantly reduced in the HUMSC group (*F*(3,20) = 53.45, *P* < 0.0001) and the HUMSC + Lav 100, 200, and 400 treatment groups (*F*(6,44) = 46.75, *P* < 0.0001) compared to the SCI group (Figure 3(a)).

There were significant differences between the HUMSC group and the HUMSC + Lav-treated groups compared to the SCI group. Cavity sizes were significantly reduced in the HUMSC (*P* < 0.05), HUMSC + Lav 100 (*P* < 0.001), and HUMSC + Lav 200 and HUMSC + Lav 400 (*P* < 0.0001) groups. No statistically significant differences were found in cavity volume between the HUMSC group and the HUMSC + Lav 100 or HUMSC + Lav 200 groups.

In addition, application of one-way ANOVA showed significant differences between the sham, HUMSC, HUMSC + Lav, and control groups in the number of ventral horn motor neurons (*F*(6,45) = 17.45, *P* < 0.0001). Application of Bonferroni's post hoc multiple-comparisons test, as well as Bartlett's test for equal variances, revealed significant increases in the number of ventral horn motor neurons in the HUMSC (*P* < 0.05), HUMSC + Lav 100 (*P* < 0.05), and HUMSC + Lav 200 and HUMSC + Lav 400 (*P* < 0.0001) groups compared to the SCI group (Figure 3(b)). No significant differences were observed between the HUMSC group and the HUMSC + Lav 100 and HUMSC + Lav 200 groups, but there were significant differences between the HUMSC and HUMSC + Lav 100 groups and the HUMSC + Lav 400 group (*P* < 0.001).

### 3.5. Effects of* L. angustifolia* Extract on GFAP Expression after SCI Treatment with HUMSCs from Wharton's Jelly

**Figure 4:**
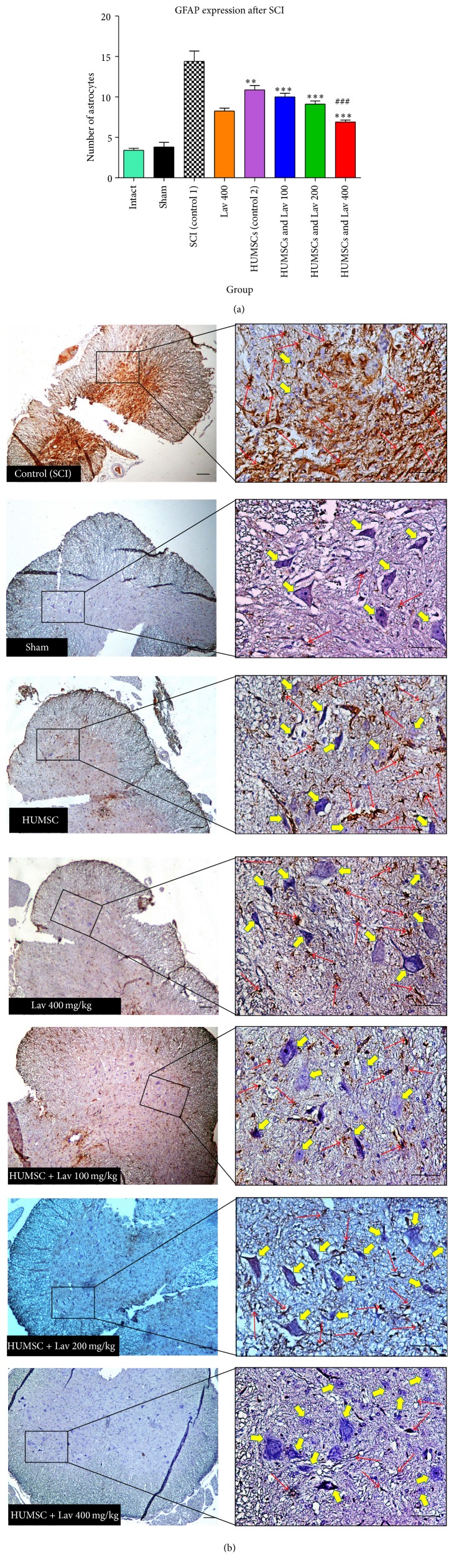
Administration of* L. angustifolia* decreased GFAP expression in the rat spinal cord contusion model transplanted HUMSCs. Intraperitoneal administration of* L. angustifolia* extract daily for 14 consecutive days postinjury significantly decreased the levels of GFAP expression in the rat spinal cord contusion model. Data are represented as the mean ± S.E.M. (a) ^*∗*^, ^*∗∗*^, and ^*∗∗∗*^ show significant differences between HUMSCs, HUMSCs + Lav 100, 200, and 400, and SCI (Control 1) (*P* < 0.05, *P* < 0.0001, and *P* < 0.0001, resp.). ^###^ shows significant differences between HUMSCs and HUMSCs + Lav 400 (*P* < 0.0001) (Per 35625 *μ*m^2^). (b) Transverse section of spinal cord showing the ventral horn gray matter at the T12-L1 level for all groups on day 56 GFAP-stained images. Yellow arrows indicate VHMNs. Red arrows indicate the GFAP astrocytes. Decreased GFAP astrocytes and increased VHMNs are evident. Bar in 40X = 100 micrometer and bar in 200X = 50 micrometer. (ECLIPSE 5Oi microscope).

**Figure 5:**
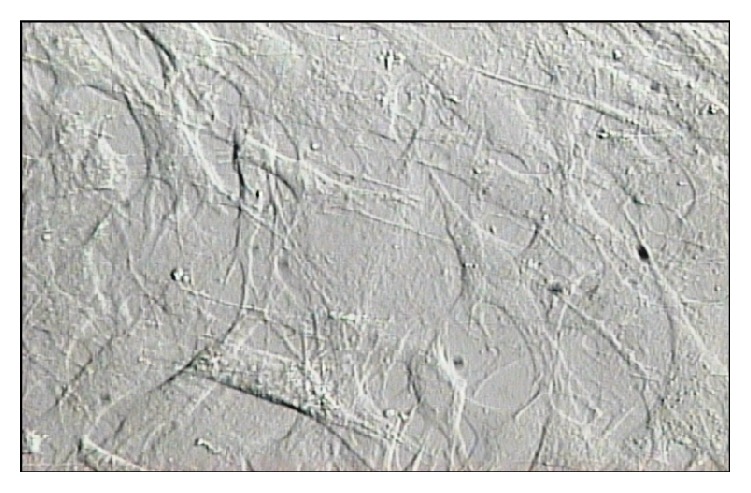
Mesenchymal Wharton's jelly stem cell. Dissociated mesenchymal Wharton's jelly stem cells were dispersed in 10% FBS-DMEM and counted under a microscope with the aid of a hemocytometer. The mesenchymal cells were then used directly for cultures or stored in liquid nitrogen for later use. 24 hours after injury marked HUMSCs (3 × 10^5^ cells/*μ*L) with BrdU in 9 *μ*L of normal saline were sucked in to a Hamilton syringe and were injected slowly at a rate of 0.25 *μ*L/min by microinjector to 3 separate places and transplanted into the three sites of lesion area (epicenter, distal, and proximal) at a depth of 1.2 mm.

Strong immunostaining for GFAP was demonstrated in the control group (Figure 3(a)); however, this activation was significantly attenuated in the HUMSC and HUMSC + Lav groups (*F*(9,66) = 25.57, *P* < 0.0001). Application of Bonferroni's post hoc multiple-comparisons test, as well as Bartlett's test for equal variances, revealed significantly decreased GFAP expression in the HUMSC (*P* < 0.001) group and in the HUMSC + Lav 100, HUMSC + Lav 200, and HUMSC + Lav 400 groups (all *P* < 0.0001) compared to the SCI group (Figure 3(b)). No statistically significant difference was found in GFAP expression between the HUMSC + Lav 200 and HUMSC + Lav 400 groups, but there was significantly decreased GFAP expression in the SC + Lav 400 group compared to the HUMSC-treated groups (*P* < 0.0001) (Figure 4).

## 4. Discussion

**Figure 6:**
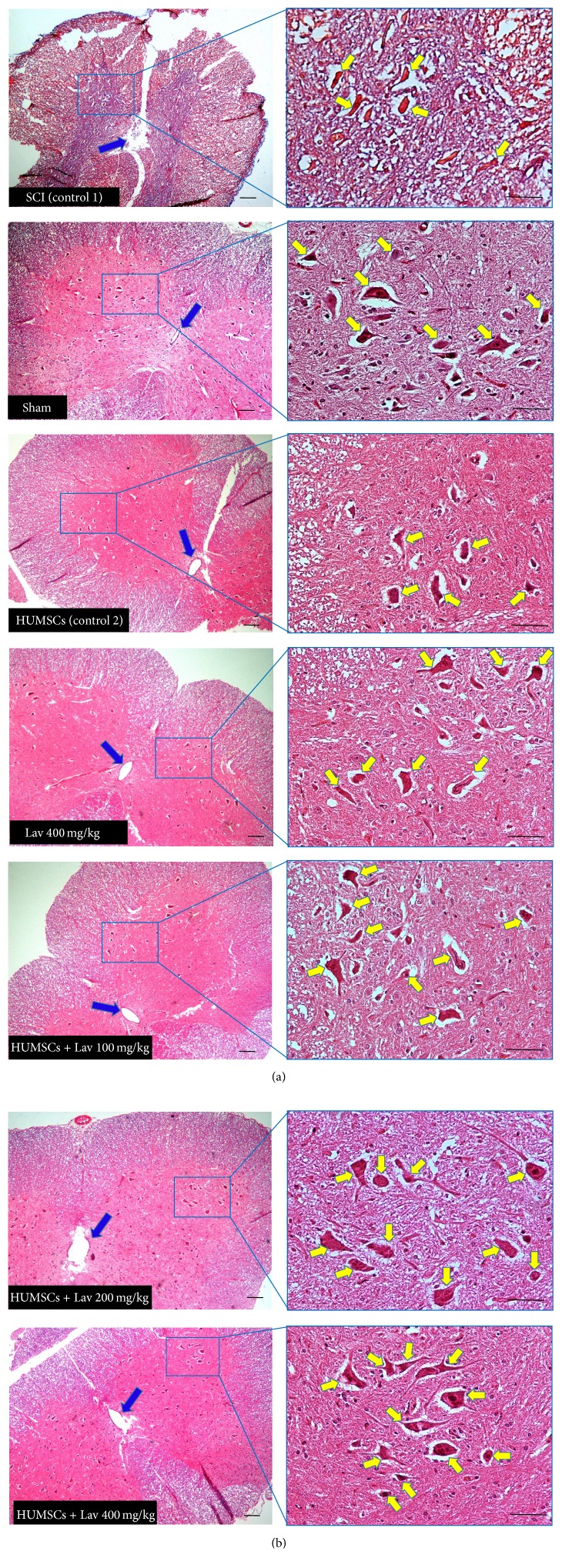
Stem cell therapy and administration of* L. angustifolia* improved histomorphological evaluation results in the rat spinal cord contusion model. Transverse section of spinal cord showing the ventral horn gray matter from spinal cord at the level of T12-L1 of all groups which evaluated in this study at day 56. H&E staining showed shrinkage and decrease of ventral horn motor neurons of HUMSCs in compare with control 1 (SCI) (*P* < 0.0001) and HUMSCs + Lav 100, 200, and 400 mg/Kg/day in comparison with HUMSCs. Yellow arrows illustrating the ventral horn motor neurons and blue arrows show central canal. HUMSCs therapy improved the number and shape of central canal and* L. angustifolia* extract potentiated this effect. Bar in 40X = 100 micrometer and bar in 200X = 50 micrometer. (ECLIPSE 5Oi microscope).

Some studies have shown unsuccessful effects of stem cell transplantation for SCI treatment [29], but ample evidence demonstrates that stem cells are effective in such treatments. Therefore, controversy exists, as some reviewers believe that stem cells have little effect on treating SCI [30]. However, stem cell therapy is administered to SCI patients by surgeons in operating rooms throughout the world [21, 30]. We decided to potentiate the effects of transplanted stem cells by adding a neuroprotective and effective drug to induce curative effects in SCI-induced rats.* L. angustifolia* has neuroprotective and neurotrophic effects [31], including enhancement of functional recovery [31], suggesting that it has a therapeutic effect on neurodegenerative disease [28, 31, 32] (Figure 6). Our previous research demonstrated that administration of* L. angustifolia* extract itself improved behavioral, sensory, and cellular outcomes in a rat SCI model. In the present study, we evaluated the therapeutic potential of* L. angustifolia* for SCIs treated with HUMSCs in a conventional animal model. Intraperitoneal* L. angustifolia* improved motor function following contusion SCIs treated with HUMSCs. Thus, this is the first report demonstrating that administration of* L. angustifolia* extract itself potentiates behavioral and cellular outcomes in SCI rats treated with mesenchymal Wharton's jelly stem cells. Our findings are consistent with Yang's results, which successfully demonstrated improvements in locomotion and axonal regeneration in the corticospinal tract, even after complete transection of the rat spinal cord [33].

SCI involves a variety of neurochemical, cellular, and molecular events, including calcium overload [34], extracellular accumulation of glutamate [3], and induction of oxidative stress [5, 35]. Increased oxidative stress after spinal cord trauma can lead to secondary processes, such as impaired activity of membrane enzymes [35, 36] and overexpression of inflammatory mediators, which potentiates secondary injuries to the spinal cord via a variety of processes, such as activation of microglia and stimulation of astrocyte proliferation [6, 37].

It has been demonstrated that administration of* L. angustifolia* extract could alleviate the extracellular accumulation of glutamate [38] and could decrease oxidative stress [31, 32]. Protection against the progression of secondary injury to spinal cord neurons appears to be one of the most effective therapeutic strategies for limiting tissue injury and improving the outcome of spinal cord trauma [4]. As neuroprotection could preserve neurologic function by preventing cell death, one of the most important roles of* L. angustifolia* could be its neuroprotective effects. We believe that calcium-calmodulin may play an important role in the neuroprotective effects of* L. angustifolia* [24].

Our findings are consistent with Yang's results, which showed improvement in locomotion and axonal regeneration in the corticospinal tract even after complete transection of the rat spinal cord [33]. However, our results showed that Lav 400 mg/kg can also potentiate the curative effects of stem cell transplantation in SCI rats.

Due to* L. angustifolia*'s antimicrobial, anti-inflammatory, and analgesic properties, it seems that it could prevent wound infections and play a role in reducing pain by lowering inflammation [39]. This effect of* L. angustifolia* should make it easier to induce the therapeutic effects of HUMSCs.

Certain growth factors, such as epidermal growth factor (EGF), insulin-like growth factor (IGF), platelet-derived growth factor (PDGF), and fibroblast growth factor (FGF), regulate cellular proliferation, differentiation and migration, and the synthesis of extracellular matrix proteins, as well as angiogenesis during wound healing [40, 41]. There are some reports that lavender oil reduces scar tissue in wound healing [42]; although this needs further investigation, it may reduce axonal scarring in SCI.

According to another study,* L. angustifolia* oil resulted in the highest EGF and FGF-2 reactions, from which it was concluded that* L. angustifolia* was effective in the stimulation of reepithelialization and granulation for tissue formation [43]. HUMSCs have been used in animal models and clinical trials for the treatment of many diseases, such as myocardial infarction, graft-versus-host disease, stroke, and SCI [44], and have demonstrated paracrine, immunomodulatory, anti-inflammatory, and antiapoptotic effects [45]. HUMSCs can migrate and secrete a variety of cytokines in injured tissues, including IGF, brain-derived neurotrophic factor (BDNF), vascular EGF, granulocyte-macrophage colony stimulating factor (GM-CSF), FGF-2, and transforming growth factor (TGF) [46]. Therefore, it is clear that the potentiation of the effects of HUMSCs in SCI animals is relevant to* L. angustifolia*, and the synergic effects of these two separate and inexpensive treatment protocols potentiate each other.

## 5. Conclusion

In conclusion, HUMSCs from Wharton's jelly improved motor function and promoted morphological improvement in a rat SCI contusion model. Due to its neuroprotective properties,* L. angustifolia* extract potentiated the motor, sensory, and cellular improvements associated with HUMSC transplantation for SCI. While further studies are needed to clarify the mechanism of action of* L. angustifolia* in SCI models, the present results suggest that* L. angustifolia* extract can potentiate the therapeutic effects of Wharton's jelly HUMSCs for treating patients with SCI.

## References

[1] Sekhon L. H. S., Fehlings M. G. (2001). Epidemiology, demographics, and pathophysiology of acute spinal cord injury. *Spine*.

[2] Coutts M., Keirstead H. S. (2008). Stem cells for the treatment of spinal cord injury. *Experimental Neurology*.

[3] Beattie M. S., Hermann G. E., Rogers R. C., Bresnahan J. C. (2002). Cell death in models of spinal cord injury. *Progress in Brain Research*.

[4] Dumont A. S., Dumont R. J., Oskouian R. J. (2002). Will improved understanding of the pathophysiological mechanisms involved in acute spinal cord injury improve the potential for therapeutic intervention?. *Current Opinion in Neurology*.

[5] Azbill R. D., Mu X., Bruce-Keller A. J., Mattson M. P., Springer J. E. (1997). Impaired mitochondrial function, oxidative stress and altered antioxidant enzyme activities following traumatic spinal cord injury. *Brain Research*.

[6] Popovich P. G., Jones T. B. (2003). Manipulating neuroinflammatory reactions in the injured spinal cord: back to basics. *Trends in Pharmacological Sciences*.

[7] Beattie M. S., Farooqui A. A., Bresnahan J. C. (2000). Review of current evidence for apoptosis after spinal cord injury. *Journal of Neurotrauma*.

[8] Chen M. S., Huber A. B., van der Haar M. E. (2000). Nogo-A is a myelin-associated neurite outgrowth inhibitor and an antigen for monoclonal antibody IN-1. *Nature*.

[9] Cai D., Qiu J., Cao Z., McAtee M., Bregman B. S., Filbin M. T. (2001). Neuronal cyclic AMP controls the developmental loss in ability of axons to regenerate. *Journal of Neuroscience*.

[10] Horner P. J., Power A. E., Kempermann G. (2000). Proliferation and differentiation of progenitor cells throughout the intact adult rat spinal cord. *The Journal of Neuroscience*.

[11] Kojima A., Tator C. H. (2000). Epidermal growth factor and fibroblast growth factor 2 cause proliferation of ependymal precursor cells in the adult rat spinal cord in vivo. *Journal of Neuropathology & Experimental Neurology*.

[12] Martens D. J., Seaberg R. M., van der Kooy D. (2002). In vivo infusions of exogenous growth factors into the fourth ventricle of the adult mouse brain increase the proliferation of neural progenitors around the fourth ventricle and the central canal of the spinal cord. *European Journal of Neuroscience*.

[13] Kojima A., Tator C. H. (2002). Intrathecal administration of epidermal growth factor and fibroblast growth factor 2 promotes ependymal proliferation and functional recovery after spinal cord injury in adult rats. *Journal of Neurotrauma*.

[14] Wang H.-S., Hung S.-C., Peng S.-T. (2004). Mesenchymal stem cells in the Wharton's jelly of the human umbilical cord. *STEM CELLS*.

[15] Taghizadeh R. R., Cetrulo K. J., Cetrulo C. L. (2011). Wharton's Jelly stem cells: future clinical applications. *Placenta*.

[16] Goudarzi M. M., Azarnia M., Kaka G., Sadraii S. H., Aghda A. K. (2013). Study of bone marrow stromal cells, nerve growth factor, and marginal on nerve regeneration in rat crushed sciatic nerve. *Journal of Mazandaran University of Medical Sciences*.

[17] Mitchell K. E., Weiss M. L., Mitchell B. M. (2003). Matrix cells from Wharton's jelly form neurons and glia. *Stem Cells*.

[18] Kaka G. R., Tiraihi T., Delshad A., Arabkheradmand J., Kazemi H. (2012). In vitro differentiation of bone marrow stromal cells into oligodendrocyte-like cells using triiodothyronine as inducer. *International Journal of Neuroscience*.

[19] Björklund A. (1993). Better cells for brain repair. *Nature*.

[20] Chao K. C., Chao K. F., Fu Y. S., Liu S. H. (2008). Islet-like clusters derived from mesenchymal stem cells in Wharton's jelly of the human umbilical cord for transplantation to control type 1 diabetes. *PLoS ONE*.

[21] Richard Winn H., Volker C. I. S., Sonntag K. H., Voll D. G. (2012). Spine. *YOUMANS Neurological Surgery*.

[22] Omidbeigi R. *Production and Processing of Medicinal Plants*.

[23] Avicenna T. b. S. A. *Canon of Medicine (Qanun dar Tib)*.

[24] Kayvan Yaghoobi G. K., Davoodi S., Ashayeri H. (2016). Therapeutic effects of *Lavandula angustifolia*. *Journal of Gorgan University of Medical Sciences*.

[25] Saadatian M., Aghaei M., Farahpour M. R., Balouchi Z. (2013). Chemical composition of lavender (*Lavandula officinallis* L.) extraction extracted by two solvent concentrations. *Global Journal of Medicinal Plant Research*.

[26] Cong Y., Abulizi P., Zhi L., Wang X., Mirensha (2008). Chemical composition of the essential oil of *Lavandula angustifolia* from Xinjiang, China. *Chemistry of Natural Compounds*.

[27] Peana A. T., De Montis M. G., Nieddu E., Spano M. T., D'Aquila P. S., Pippia P. (2004). Profile of spinal and supra-spinal antinociception of (−)-linalool. *European Journal of Pharmacology*.

[28] Peana A. T., D'Aquila P. S., Chessa M. L., Moretti M. D. L., Serra G., Pippia P. (2003). (−)-Linalool produces antinociception in two experimental models of pain. *European Journal of Pharmacology*.

[29] Jiří Růžička N. R., Hejčl A., Vetrík M. (2013). Treating spinal cord injury in rats with a combination of human fetal neural stem cells and hydrogels modified with serotonin. *Acta Neurobiologiae Experimentalis*.

[30] Winn H. R., Sonntag V. K. H., Dennis G. (2012). *YOUMANS Neurological Surgery*.

[31] Vakili A., Sharifat S., Akhavan M. M., Bandegi A. R. (2014). Effect of lavender oil (*Lavandula angustifolia*) on cerebral edema and its possible mechanisms in an experimental model of stroke. *Brain Research*.

[32] Peana A. T., Marzocco S., Popolo A., Pinto A. (2006). Linalool inhibits in vitro NO formation: probable involvement in the antinociceptive activity of this monoterpene compound. *Life Sciences*.

[33] Yang C.-C., Shih Y.-H., Ko M.-H., Hsu S.-Y., Cheng H., Fu Y.-S. (2008). Transplantation of human umbilical mesenchymal stem cells from Wharton's jelly after complete transection of the rat spinal cord. *PLoS ONE*.

[34] Dumont R. J., Okonkwo D. O., Verma S. (2001). Acute spinal cord injury: part I. Pathophysiologic mechanisms. *Clinical Neuropharmacology*.

[35] Aksenova M., Butterfield D. A., Zhang S.-X., Underwood M., Geddes J. W. (2002). Increased protein oxidation and decreased creatine kinase BB expression and activity after spinal cord contusion injury. *Journal of Neurotrauma*.

[36] Martin L. J., Liu Z. (2002). Injury-induced spinal motor neuron apoptosis is preceded by DNA single-strand breaks and is p53- and Bax-dependent. *Journal of Neurobiology*.

[37] Dumont R. J., Okonkwo D. O., Verma S. (2001). Acute spinal cord injury, part I: pathophysiologic mechanisms. *Clinical Neuropharmacology*.

[38] Büyükokuroğlu M. E., Gepdiremen A., Hacimüftüoğlu A., Oktay M. (2003). The effects of aqueous extract of *Lavandula angustifolia* flowers in glutamate-induced neurotoxicity of cerebellar granular cell culture of rat pups. *Journal of Ethnopharmacology*.

[39] Lusby P. E., Coombes A. L., Wilkinson J. M. (2006). A comparison of wound healing following treatment with *Lavandula* x *allardii* honey or essential oil. *Phytotherapy Research*.

[40] Werner S., Grose R. (2003). Regulation of wound healing by growth factors and cytokines. *Physiological Reviews*.

[41] Barrientos S., Stojadinovic O., Golinko M. S., Brem H., Tomic-Canic M. (2008). Growth factors and cytokines in wound healing. *Wound Repair and Regeneration*.

[42] Cavanagh H. M. A., Wilkinson J. M. (2002). Biological activities of lavender essential oil. *Phytotherapy Research*.

[43] Koca Kutlu A., Çeçen D., Gürgen S. G., Sayn O., Çetin F. (2013). A comparison study of growth factor expression following treatment with transcutaneous electrical nerve stimulation, saline solution, povidone-iodine, and lavender oil in wounds healing. *Evidence-Based Complementary and Alternative Medicine*.

[44] Caplan A. I. (2007). Adult mesenchymal stem cells for tissue engineering versus regenerative medicine. *Journal of Cellular Physiology*.

[45] da Silva Meirelles L., Fontes A. M., Covas D. T., Caplan A. I. (2009). Mechanisms involved in the therapeutic properties of mesenchymal stem cells. *Cytokine and Growth Factor Reviews*.

[46] Azari M. F., Mathias L., Ozturk E., Cram D. S., Boyd R. L., Petratos S. (2010). Mesenchymal stem cells for treatment of CNS injury. *Current Neuropharmacology*.

